# Novel internalin P homologs in *Listeria*

**DOI:** 10.1099/mgen.0.000828

**Published:** 2022-07-29

**Authors:** Kayla N. Conner, Joseph T. Burke, Janani Ravi, Jonathan W. Hardy

**Affiliations:** ^1^​ Department of Microbiology and Molecular Genetics, Michigan State University, East Lansing, MI, USA; ^2^​ Institute for Quantitative Health Science and Engineering, Michigan State University, East Lansing, MI, USA; ^3^​ Genomics and Molecular Genetics Undergraduate Program, College of Natural Science, Michigan State University, East Lansing, MI, USA; ^4^​ Department of Pathobiology and Diagnostic Investigation, Michigan State University, East Lansing, MI, USA; ^5^​ Department of Biomedical Informatics, Center for Health Artificial Intelligence, University of Colorado Anschutz Medical Campus, Aurora, CO, USA

**Keywords:** inlp, internalin, *Listeria*, molecular evolution, phylogeny, placenta

## Abstract

*
Listeria monocytogenes* (*Lm*) is a bacterial pathogen that causes listeriosis in immunocompromised individuals, particularly pregnant women. Several virulence factors support the intracellular lifecycle of *Lm* and facilitate cell-to-cell spread, allowing it to occupy multiple niches within the host and cross-protective barriers, including the placenta. One family of virulence factors, internalins, contributes to *Lm* pathogenicity by inducing specific uptake and conferring tissue tropism. Over 25 internalins have been identified thus far, but only a few have been extensively studied. Internalins contain leucine-rich repeat (LRR) domains that enable protein-protein interactions, allowing *Lm* to bind host proteins. Notably, other *Listeria* species express internalins but cannot colonize human hosts, prompting questions regarding the evolution of internalins within the genus *Listeria *. Internalin P (InlP) promotes placental colonization through interaction with the host protein afadin. Although prior studies of InlP have begun to elucidate its role in *Lm* pathogenesis, there remains a lack of information regarding homologs in other *Listeria* species. Here, we have used a computational evolutionary approach to identify InlP homologs in additional *Listeria* species. We found that *Listeria ivanovii londoniensis* (*Liv*) and *Listeria seeligeri* (*Ls*) encode InlP homologs. We also found InlP-like homologs in *Listeria innocua* and the recently identified species *Listeria costaricensis *. All newly identified homologs lack the full-length LRR6 and LRR7 domains found in *Lm*’s InlP. These findings are informative regarding the evolution of one key *Lm* virulence factor, InlP, and serve as a springboard for future evolutionary studies of *Lm* pathogenesis as well as mechanistic studies of *Listeria* internalins.

## Data Summary

The authors confirm all supporting data, analyses, and visualizations have been provided within the article or through supplementary data files. They are also available in our GitHub repository, https://github.com/JRaviLab/inlp_listeria. Text, figures and data are licensed under Creative Commons Attribution CC BY 4.0.

**Impact Statement**

The intracellular bacterial pathogen *Listeria monocytogenes* can breach protective barriers in the pregnant host, allowing the colonization of the placenta in pregnant people and resulting in numerous adverse pregnancy outcomes. Previous studies aimed at delineating the mechanisms behind placental colonization of *L. monocytogenes* identified a key virulence factor, internalin P (InlP). The internalin family of proteins has been studied extensively due to their conservation in the genus *Listeria* and their contribution to virulence and pathogenicity in *L. monocytogenes *. Still, many questions remain regarding the evolution of internalins and their potential roles in non-pathogenic *Listeria *. Our work addresses this gap in knowledge by (1) identifying additional InlP homologs in *Listeria *, including *L. ivanovii, L. seeligeri, L. innocua,* and *L. costaricensis *, and (2) characterizing these homologs using computational evolutionary methods to compare their primary sequences, domain architectures, and structural models. Together, our findings contribute to the field by providing insights into the evolution of one key member of the internalin family, as well as serving as a catalyst for future studies of InlP and its role in *Listeria* pathogenesis.

## Introduction

Prenatal infection remains a major public health concern. Annually, nearly 13 million infants are born prematurely worldwide, and an estimated 30% of these preterm births can be attributed to prenatal infection, although the actual number may be higher due to the subclinical nature of many prenatal infections [1]. To better detect and treat these infections to prevent adverse pregnancy outcomes, we must better understand the pathogens that cause them. *Listeria monocytogenes* is widely used in prenatal infection research due to its well-characterized life cycle and ease of use in laboratory experiments [2, 3].

The genus *Listeria* comprises 17 species, including the human pathogens *L. ivanovii* (*Liv*) and *L. monocytogenes* (*Lm*) [4]. These Gram-positive facultative intracellular bacterial pathogens are the most typical causative agent of listeriosis in humans [4, 5]. While relatively rare, listeriosis can result in severe morbidity and mortality in immunocompromised individuals [5, 6]. Pregnant people are particularly at risk for listeriosis, as *Lm* can colonize the placenta and cause adverse pregnancy outcomes, such as preterm birth, neonatal meningitis, miscarriage, and stillbirth [5]. They are approximately 10 times more likely to contract listeriosis compared to their immunocompetent counterparts, comprising 17% of all annual cases of listeriosis [7, 8]. *Lm* employs several virulence factors that aid in its invasion of various host niches and the breach of protective host barriers [5, 6]. Previous studies have addressed the roles of various *Lm* virulence factors, such as ActA, internalin A (InlA), and internalin B (InlB) in the context of pregnancy [9–11]. Faralla *et al*., followed up with two studies focusing on internalin P (InlP), a key virulence factor for the invasion of the placenta [12, 13].

Typically, listeriosis begins with the consumption of contaminated food items. Once in the digestive system, *Lm* uses several virulence factors, including the internalins, to colonize gut epithelial cells and spread throughout the host [5, 6, 14]. Internalins contribute to this spread by conferring tissue tropism; for example, InlA binds E-cadherin on gut epithelial cells, while InlB binds C-Met expressed by hepatocytes [14]. These interactions are enabled by leucine-rich repeat (LRR) domains found in all internalins [14, 15]. LRR domains are found in an array of functionally diverse proteins across the domains of life. LRRs are found in ribonuclease inhibitors in humans and pigs, connectin in *Drosophila*, adenylate cyclase in *Saccharomyces*, transmembrane kinase I in *Arabidopsis thaliana*, and various virulence factors in pathogens such as *Yersinia pestis* and *Lm* [15, 16]. Internalins may have as few as 4 (InlG) or as many as 14 (InlA) LRR domains [5, 14]. Notably, internalins and other virulence factors are relatively well-conserved across the genus, including species that are considered non-pathogenic to humans, such as *L. seeligeri* and *L. innocua* [4, 17, 18]. While the details remain unclear, differences in pathogenicity have been attributed to minor genetic variations and differences in the expression of virulence factor genes [19]. The precise roles of the various internalin genes in *Listeria* and their evolutionary relationships remain critical open questions in *Listeria* biology.

Internalin P (InlP) is an *Lm* virulence factor known to enhance placental colonization in the pregnant host. This is likely accomplished by enabling *Lm* to transcytose through the basal membrane underlying the syncytiotrophoblast, the protective outer layer of placental cells that serves as a barrier between maternal and foetal blood [12]. Further characterization revealed that InlP encompasses nine LRR domains and binds the human protein afadin, which is a nectin-like protein found in cell-cell junctions and is thought to play a significant role in cellular adhesion [13].

Initial InlP studies identified a structural homolog of InlP, Lmo2027, in *Lm*, but information regarding InlP homologs in other *Listeria* species has been incomplete [12]. In this study, we used comparative genomics and protein sequence-structure-function analyses to identify InlP homologs in the genomes of *L. seeligeri* (*Ls*), *L. ivanovii londoniensis* (*Liv*), *L. innocua* (*Lin*) and *L. costaricensis* (*Lc*). The bioinformatic analysis presented here serves as a springboard for future studies of *Listeria* evolution and pathogenesis pertaining to the internalin protein family, including its ability to colonize the human placenta.

## Methods

### Identification of inlp homologs

To identify InlP*_Lm_* homologs across evolutionary lineages, we submitted the InlP*_Lm_* amino acid sequence (accession: WP_014601135.1) to MolEvolvR [20] (http://www.jravilab.org/molevolvr). The query returned hits for homologous proteins across bacterial phyla. While many species carried homologous proteins (*e.g. Nostoc* spp. and *Beggiatoa leptomitoformis *), we chose to filter out hits with low similarity and divergent domain architectures and genomic contexts for our detailed study; we thus focused on the genus *Listeria* (including 45 530 *L *. *monocytogenes *, 740 *L *. *innocua *, 169 *L *. *seeligeri *, 44 *L *. ivanovii*,* and 1 *L *. *costaricensis* genomes). Within this dataset, we selected the hits with the highest percentage similarity and unique domain architectures as representative homologs for further analysis. Accession numbers provided by MolEvolvR were used to query the National Center for Biotechnology Information (NCBI) RefSeq protein database for corresponding nucleotide sequences, locus tags, and isolate source (where available) for homologous genes [21]. BioCyc [22] (https://biocyc.org) and NCBI RefSeq [21] protein databases were used to identify genomic contexts (neighbouring genes).

### Calculation of percentage identity and percentage similarity

Percentage identity and percentage similarity values for predicted homologs were provided by MolEvolvR [20] (Table S1, available in the online version of this article; https://github.com/jravilab/inlp_listeria). Nucleotide sequences for *inlP* in *L. monocytogenes *, *L. ivanovii londoniensis *, *L. seeligeri* and *L. costaricensis* were aligned using Clustal Omega [23] (https://www.ebi.ac.uk/Tools/msa/clustalo/), and the resulting alignments were submitted in FASTA format to the Sequence Manipulation Suite [24] (SMS; http://www.bioinformatics.org/sms2) to calculate percentage nucleotide identity. The homologue similarity and identity matrix was generated using MatGAT2.01 with the BLOSUM 62 matrix and default options [25].

### Multiple sequence alignment, phylogenetic trees and protein models

**Fig. 1.**
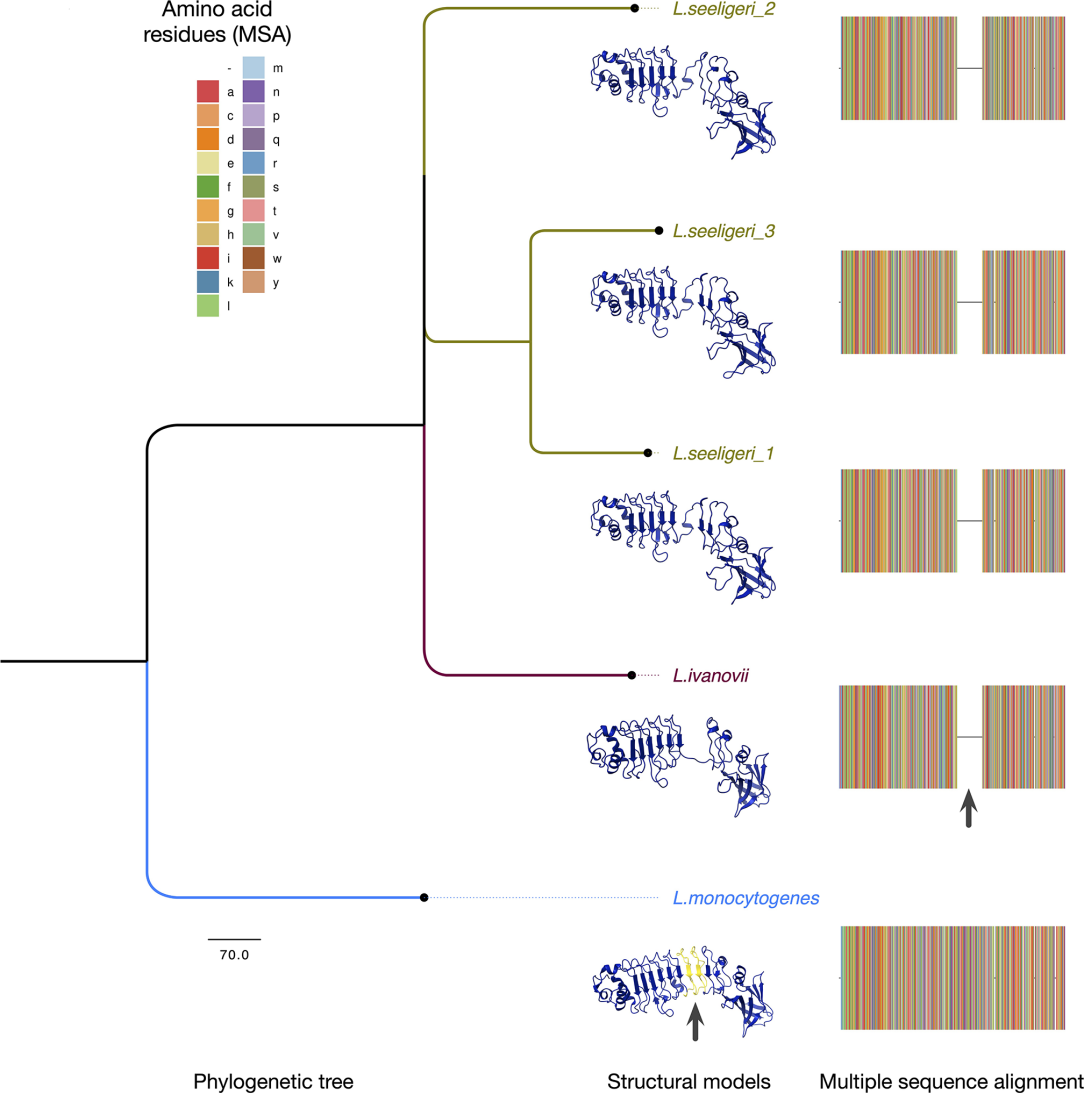
Phylogeny and structure models of InlP and representative Homologs. A phylogenetic tree was generated using the amino acid sequences of InlP homologs in *L. monocytogenes* (blue), *L. ivanovii londoniensis* (purple) and *L. seeligeri* (green). Three-dimensional models were generated using SWISS-MODEL with the crystal structure for InlP*_Lm_* (PDB: 5hl3) as a template and then visualized using ChimeraX. Multiple sequence alignments were generated using MolEvolvR and illustrate the complete LRR6 and LRR7 insertion present in InlP*_Lm_* (inserted motif is highlighted in yellow in the 3D model in a backdrop of a blue protein structure model; also indicated with the arrow). The legend shows the colours of the amino acid residues indicated in the multiple sequence alignment. The height of the MSA (for each of the five sequences) has been increased to show the colours more distinctly, and to highlight the missing motif indicated by the arrow.

**Fig. 2.**
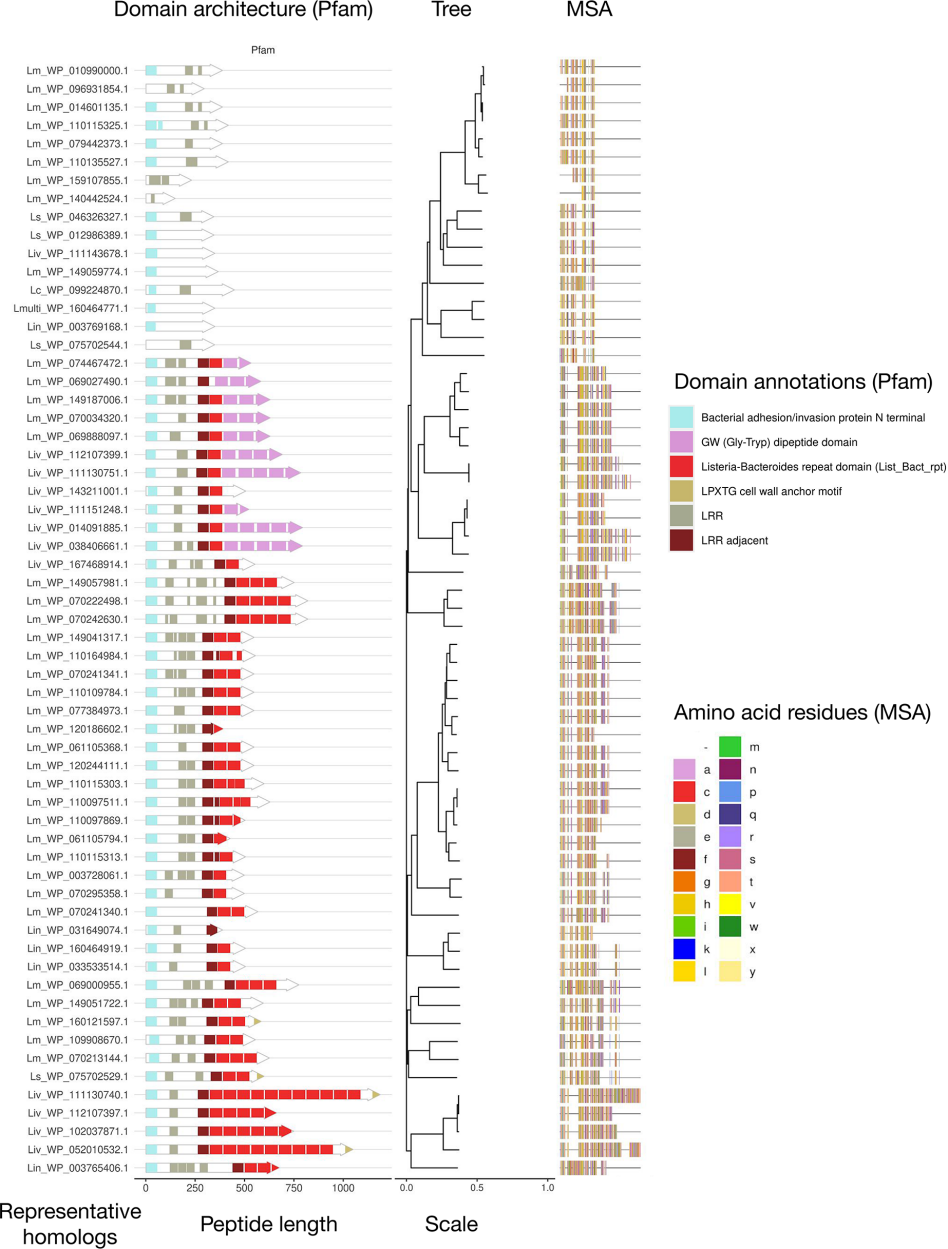
Phylogeny and domain architectures of putative internalin P Homologs. A multiple sequence alignment and phylogenetic tree were generated using the amino acid sequences of putative InlP homologs identified using MolEvolvR and five *Listeria* InlP starting points (see the Methods section). The phylogeny of InlP-like proteins (with branch lengths marked using *d*_N_/*d*_S_ ratio with the ggtree package) has been overlaid with their domain architectures (generated using MolEvolvR, showing only Pfam domain architectures). The two legends show the colours of the amino acids indicated in the multiple sequence alignment (corresponding to the MSA, right panel) and the Pfam domain annotations (corresponding to the domain architecture, left panel). The arrow lengths and the overall coloured MSA segments correspond to the relative lengths of each of the InlP-like proteins.

Multiple sequence alignments for homologous amino acid and nucleotide sequences were generated using Kalign [26] and visualized using JalView (version 2.11.1.4) [27] with default parameters. Multiple sequence alignments in Figs 1 and 2 were generated using the msaplot function in the ggtree R package with default parameters [28]. Neighbour-joining trees were constructed using the ape package in R [29]. Domain architectures were determined using Interproscan with default parameters. Three-dimensional protein models were produced using SWISS-MODEL with the *L. monocytogenes* internalin P crystal structure (PDB: 5hl3) as a template and visualized using ChimeraX [30–32]. All data, analyses and visualizations for InlP *Listeria* homologs are available at https://github.com/jravilab/inlp_listeria.

## Results

### *L. ivanovii londoniensis* and *L. seeligeri* encode internalin P homologs

**Fig. 3.**
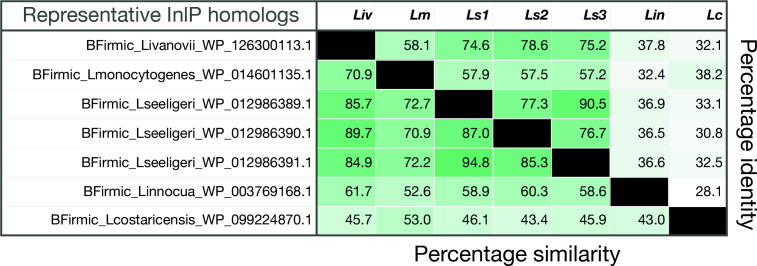
Percentage similarity and identity of internalin P Homologs in representative *Listeria* species. Percentage similarity and percentage identity were calculated for the amino acid sequences for representative internalin P homologs from five *Listeria* species, *L. monocytogenes, L. ivanovii, L. seeligeri, L. costaricensis* and *L. innocua *. Matrix showing similarity and identity values was generated using MatGAT2.01 with the BLOSUM 62 matrix and default options were selected.

To begin investigating the evolutionary conservation of the *L. monocytogenes* internalin P (InlP*_Lm_*), we started with an extensive homology search and protein characterization of InlP-like proteins in diverse lineages across the tree of life using MolEvolvR [20] (http://jravilab.org/molevolvr). Most homologs were only present within the genus *Listeria *. To further ensure that all homologs are being identified, we picked other representative InlP homologs from *L. ivanovii* and *L. seeligeri* as new starting points for our homology search and characterization (using MolEvolvR [20]; see the Methods section). We found several hits in our multi-start search, including proteins that contain transmembrane domains, resembling InlB rather than InlP (Fig. 2). Other hits carried neither the signature LRR (Fig. 2) nor internalin_N Fig. S1 domains characteristic of internalins. Therefore, we restricted our full set of homologs to InlP-like proteins, resulting in 64 representative proteins with distinct domain architectures from each *Listeria* species, including *L. monocytogenes *, *L. seeligeri *, *L. ivanovii *, *L. innocua,* and *L. costaricensis* (Figs 2 and S1, Table S1. Homologs from *L. seeligeri* (*Ls*) and *L. ivanovii* (*Liv*) showed >65 % amino acid similarity compared to InlP*_Lm_*, while homologs from *L. innocua* (*Lin*) and *L. costaricensis* (*Lc*) showed 52.6 and 53 % similarity, respectively (Fig. 3). We found that several homologs lacked predicted signal peptide domains, suggesting that they are not secreted like InlP; this was further corroborated by the presence of predicted transmembrane LPXTG motifs, which indicates that these homologs are more likely to be membrane-anchored InlB-like proteins rather than secreted InlP homologs (Figs 2 and S1). Investigation of the InlP-like proteins in *Lc and Lin* showed that it is unlikely that they are functional InlP homologs (discussed below). Further investigation of domain architectures and genomic contexts of putative homologs ultimately revealed one InlP homolog encoded within the *L. ivanovii londoniensis* genome and three InlP paralogs encoded by *L. seeligeri* (discussed below). Here, we refer to these homologs as InlP*_Lm_*, InlP*_Li_*, InlP*_Ls1_*, InlP*_Ls2_* and InlP*_Ls3_*, respectively, to indicate species and gene order.

We determined the similarity of InlP homologs in *Liv* and *Ls* at the nucleotide and amino acid levels towards functional characterization. The *inlP* gene in *L. ivanovii londoniensis* (*inlP_Liv_*), as well as the three *L. seeligeri* paralogs (*inlP_Ls1_*, *inlP_Ls2_*, and *inlP_Ls3_*), shared ~70% identity with the *inlP_Lm_* gene and ~52–65 % identity at the amino acid level when compared to InlP*_Lm_* (Fig. S2). However, the newly identified homologs in *L. ivanovii londoniensis* and *L. seeligeri* shared much higher percentage amino acid similarity with InlP*_Lm_* — InlP*_Liv,_* InlP*_Ls1_* and InlP*_Ls3_* showed ~70% similarity to InlP*_Lm_* (Fig. 3). Notably, the flanking *L. seeligeri* paralogs, InlP*_Ls1_* and InlP*_Ls3_*, were more similar to each other than to the third paralog or to I nlP*_Lm_* (Fig. 3). To further investigate these new *Listeria* InlP proteins, we next explored their genomic neighbourhoods.

**Fig. 4.**
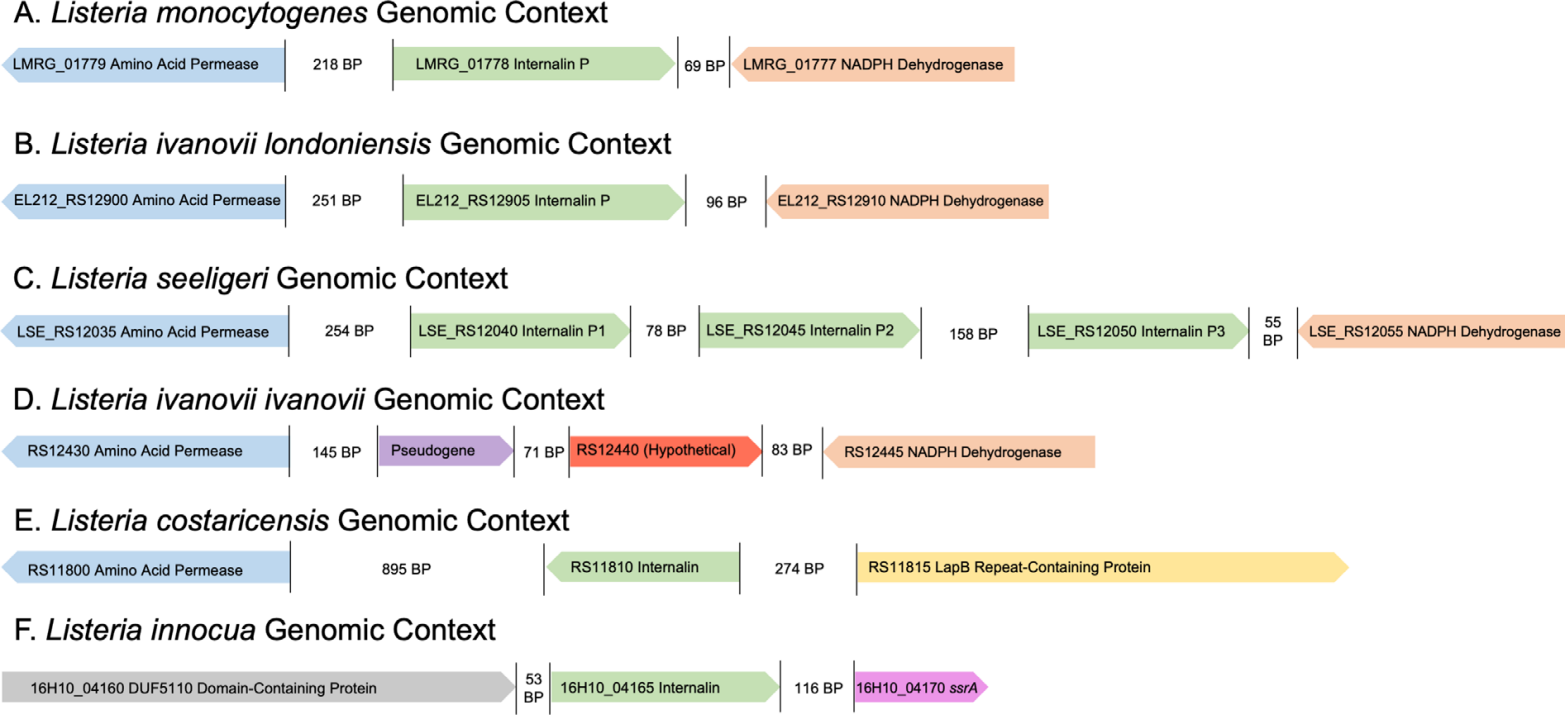
Genomic context of newly identified internalin P gene homologs. Genes homologous to the *L. monocytogenes inlP* (**a**) were identified in various other *Listeria* species. Gene order was maintained in *L. monocytogenes *, *L. seeligeri* and *L. ivanovii londoniensis *. Notably, *L. seeligeri* encodes three copies of the *inlP* gene. All homologs categorized as ‘true’ homologs were flanked upstream by an amino acid permease gene (blue) and downstream by an NADPH dehydrogenase gene (orange). *L. ivanovii ivanovii* contains a pseudogene (purple) and an uncharacterized gene encoding a hypothetical protein (red) in this region. Putative homologous genes in *L. costaricensis* and *L. innocua* did not mirror the genomic neighbourhoods seen in the other *Listeria* species. All *inlP* homologs are represented in green. Genomic context was determined using RefSeq genomic records and the BioCyc genome browsers for each species (see the Methods section).

Because the genus *Listeria* maintains a high degree of synteny across species, we investigated the genomic contexts of identified homologs compared to the InlP*_Lm_* gene, which is flanked upstream by an amino acid permease gene and downstream by an NADPH dehydrogenase gene (Fig. 4a). We hypothesized that functional homologs of InlP*_Lm_* would be flanked by these same genes in other *Listeria* species. We, therefore, determined the genomic neighbourhoods of *inlP* homologs in *L. ivanovii londoniensis, L. ivanovii ivanovii*, *L. seeligeri, L. costaricensis,* and *L. innocua* (see the Methods section). We found that the *inlP* genes in *L. ivanovii londoniensis* and *L. seeligeri* were flanked upstream by an amino acid permease gene and downstream by an NADPH dehydrogenase gene mirroring the *L. monocytogenes* genomic context, suggesting that the identified homologs are likely true homologs of the *inlP* gene (Fig. 4). Interestingly, we found that the gene encoding the InlP-like protein in *Lc* was flanked upstream by an amino acid permease and downstream by a gene encoding a LapB repeat-containing protein, inconsistent with the genomic neighbourhoods seen in other *Listeria* species (Fig. 4e). Also inconsistent with other genomic neighbourhoods, the InlP-like protein in *Lin* was flanked upstream by a DUF5110-containing protein-encoding gene and downstream by the *ssrA* gene (Fig. 4f). To quantify the similarity of the flanking genes in *L. ivanovii* londoniensis, *L. seeligeri* and *L. monocytogenes *, we calculated their pairwise similarity. At the amino acid level, the products of these flanking genes had >95 % similarity to *L. monocytogenes *. Notably, while *L. ivanovii londoniensis* encoded an *inlP* homolog in this region, *L. ivanovii ivanovii* did not (Fig. 4). Additionally, while *L. ivanovii londoniensis* only encoded one copy of *inlP* (EL212_RS12905; *inlP_Li_*), *L. seeligeri* encoded three copies (LSE_RS12040, LSE_RS12045 and LSE_RS12050; *inlP_Ls1,_ inlP_Ls2_* and *inlP_Ls3_*, respectively) (Fig. 4).

### Internalin P homologs in *L. ivanovii* and *L. seeligeri* lack the full-length LRR6 and LRR7 domains found in *L. monocytogenes* InlP

**Fig. 5.**
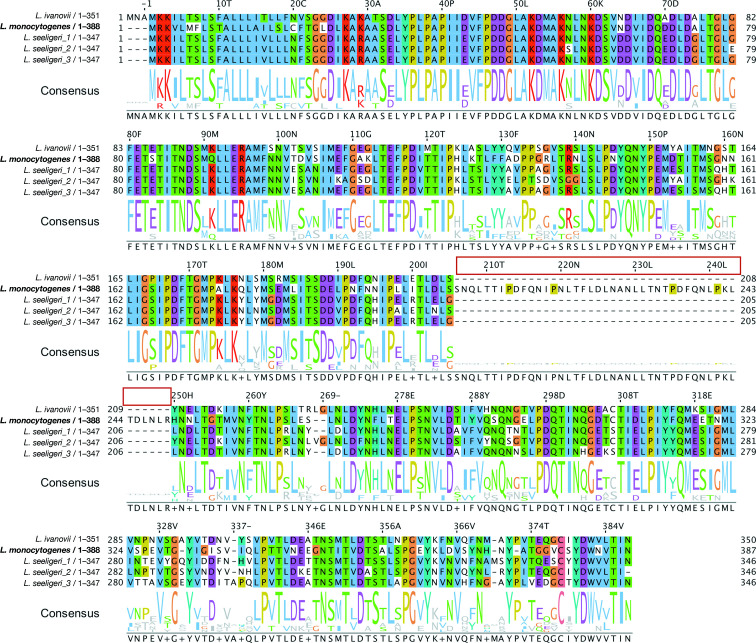
Multiple sequence alignment of internalin P homologs. Amino acid sequences for identified internalin P homologs in *L. monocytogenes *, *L. ivanovii londoniensis,* and *L. seeligeri* were aligned using Kalign and visualized using Jalview. Below the alignment is the consensus sequence for the four homologs. The red box indicates the insertion present only in the InlP*_Lm_* homolog. The *L. monocytogenes* homolog is used as the reference InlP protein (for residue numbering).

To delineate the evolution of InlP within *Listeria *, we generated a multiple sequence alignment (Fig. 5) and constructed a phylogenetic tree of the homologs (Fig. 1). While we observed several amino acid substitutions throughout the length of the proteins, the most striking difference between InlP*_Lm_* and its homologs was in the leucine-rich repeat (LRR) regions — a partial lack of LRR6 and complete lack of LRR7 — in the *L. ivanovii* and *L. seeligeri* homologs (Fig. 1). Additionally, we noted a lack of conservation in a previously described calcium-binding loop [10] present in InlP*_Lm_* (amino acid residues 132–135; Fig. 1). This observation was of particular interest since this calcium-binding loop might play a role in protein signalling or stabilization of protein-protein interactions between InlP*_Lm_* and host afadin. To better visualize the structural differences in these homologs, we generated models of InlP*_Li_*, InlP*_Ls1_*, InlP*_Ls2_*, and InlP*_Ls3_* based on the previously resolved crystal structure of InlP*_Lm_* (Fig. 1; see the Methods section). These models illustrate the similarity in the overall structure of the five homologous proteins, and the lack of LRR7 and full-length LRR6 are visible in *Li* and *Ls* homologs (Fig. 1; green/yellow regions). Additionally, the calcium-binding loop region is discernible in all five homologous proteins but appears to be structurally diverse in *L. ivanovii* and *L. seeligeri* homologs.

In summary, we have discovered novel internalin P homologs in *Listeria *, traced their evolution and uncovered potential functional implications pertaining to heterogeneity in key InlP domains. All InlP homolog data (along with characterizations in terms of domain architectures and modelling) are available at https://github.com/jravilab/inlp_listeria.

## Discussion

While previous studies have addressed many of the physical and mechanistic properties of InlP, the conservation of the *inlP* gene within or outside of the genus *Listeria* remains incompletely characterized. Here, we have provided insight into internalin P in other *Listeria* species aside from *L. monocytogenes* that will drive future mechanistic studies of InlP, as well as evolutionary studies of *Listeria* pathogenesis pertaining to the internalins.

First, we analysed the InlP amino acid sequence with MolEvolvR [20] to retrieve homologous proteins across evolutionary lineages. MolEvolvR [20] is a powerful new bioinformatic web application to characterize protein families using molecular evolution and phylogeny (http://jravilab.org/molevolvr). The MolEvolvR [20] InlP search returned a list of potential homologs, including those found in *Listeria* species. In this paper, we focus on homologs in *Listeria *, since these species carried the classic internalin and LRR domains. Using MolEvolvR [20], we identified InlP-like proteins in *L. innocua *, *L. seeligeri *, *L. ivanovii,* and *L. costaricensis *; only homologs in *L. seeligeri* and *L. ivanovii* londoniensis expressed an amino acid percentage similarity value >65 %. We found that the lower identity proteins are more likely to be internalin B homologs based on their sequence, domain architecture, and structure.

To determine whether the newly identified proteins were true homologs of InlP*_Lm_*, we explored their domain architectures and genomic context. Consistent with the synteny observed in *Listeria* genomes, we found that the *inlP* domain architectures and genomic neighbourhoods were highly conserved in *L. ivanovii londoniensis* and *L. seeligeri *, but not in *L. ivanovii ivanovii *, *L. innocua *, or *L. costaricensis *. While it is possible that these species could encode *inlP* homologs elsewhere in their genomes, it seems unlikely considering their domain architectures and lower conservation in sequence compared to other homologs. It is more likely that the homologous proteins identified in *Lc* and *Lin* are independent of InlP, but in the same class of small, secreted internalins that encompasses InlP, InlC, and InlH, among others [14].

Notably, we found that *L. ivanovii londoniensis* encoded a functional homolog for *inlP* while *L. ivanovii ivanovii* did not; *L. ivanovii ivanovii* encoded a pseudogene instead (Fig. S3). Historically, *L. ivanovii londoniensis* and *L. ivanovii ivanovii* have been distinguished biochemically [33]. Recently, Hupfeld *et al.,* noted that the two subspecies could also be distinguished based on bacteriophage susceptibility: *L. ivanovii ivanovii* strains are sensitive to bacteriophages, while *L. ivanovii londoniensis* strains encode a type II-A CRISPR-Cas system, rendering them resistant to many phages [34]. Our finding that only *L. ivanovii londoniensis *, and not *L. ivanovii ivanovii *, encodes the *inlP* gene provides another avenue for distinguishing between these two subspecies and could be beneficial to public health laboratories seeking to differentiate between them among clinical and food isolates. Additionally, because the evolution of virulence factors in *Listeria* remains mysterious, the specific presence of *inlP* in subspecies such as *ivanovii londoniensis* and its absence in *ivanovii ivanovii* may provide clues as to how *Listeria* evolves the ability to infect different cells and tissues.

Since *L. ivanovii* has been implicated in human and animal placental infection, it was not entirely surprising to find that it encoded the gene for InlP, an internalin known to enhance placental colonization. It was surprising, however, to find three copies of the *inlP* gene in *L. seeligeri *, since it has not been significantly indicated in human or animal pathogenesis [35]. Our analyses suggested that InlP*_Ls2_* was the most similar to InlP*_Lm_* and InlP*_Liv_*. It is possible that this paralog (InlP*_Ls2_*) is the ancestral one, and InlP*_Ls1_* and InlP*_Ls3_* resulted from subsequent duplication events. The presence of multiple paralogs within *L. seeligeri* suggests that InlP could have alternative functions apart from enhancing placental colonization. *Listeria* species are frequently found in environmental isolates, as they readily reside in soil. It is possible that InlP provides a fitness advantage in this environment.

One of the main questions resulting from the discovery of InlP homologs centres on the evolutionary timeline of the genus *Listeria *: which InlP came first? Our discovery that InlP*_Liv_*, InlP*_Ls1_*, InlP*_Ls2_*, and InlP*_Ls3_* do not contain the full-length LRR6 and LRR7 domains found in InlP*_Lm_* begins to offer potential answers to this question. It is plausible that *L. monocytogenes *, *L. seeligeri,* and *L. ivanovii londoniensis* shared a common ancestor that passed down the *inlP* gene, and a subsequent insertion event in *L. monocytogenes* led to the full-length InlP containing LRR6 and LRR7. Conversely, it is likely that *L. monocytogenes* carries the ancestral copy of InlP (InlP*_Lm_*); the full-length *inlP* gene could have undergone a deletion resulting in the loss of LRR6 and LRR7 in InlP*_Liv_* and InlP*_Ls_*, although it is less likely to observe several deletion events as against a single insertion event. Future studies on the evolution of the genus *Listeria* and the larger family of internalin proteins will be required to answer this question more rigorously and determine their possible links to pathogenicity.

An additional structural difference noted between newly identified InlP homologs resides in the Ca^2+^-binding loop of LRR3. Previously, this loop has been hypothesized to play a role in InlP signalling, activation, or stabilization in complex with its binding partner afadin [13]. Structural heterogeneity is visible in the Ca^2+^ regions of InlP homolog models; InlP homologs in *Liv* and *Ls* appear to have more open loops compared to InlP*_Lm_*. The ability of these loops to bind calcium, and their relative binding affinities will be an important avenue for future investigation, especially as more details regarding the function and regulation of InlP*_Lm_* come to light.

Recent studies made several fundamental discoveries regarding the physical and mechanistic properties of InlP*_Lm_* and its activity in the placenta, but many questions remain unanswered [12, 13]. The discovery of InlP homologs in *L. ivanovii londoniensis* and *L. seeligeri *, two species that have not been substantially implicated in cases of placental infection, is compelling. Future studies will investigate the activity of these homologs to determine whether they bind afadin and if they are able to enhance placental colonization of *L. monocytogenes* as well as endogenous InlP. Further, structural differences between these homologs suggest potential binding sites for the InlP-afadin interaction, which has not been resolved to date.

In summary, we report that *L. ivanovii londoniensis* and *L. seeligeri* encode homologs for the *L. monocytogenes* virulence factor InlP. Identified homologs in all three species are housed within similar genomic neighbourhoods, flanked by the same housekeeping genes upstream and downstream; further, *L. seeligeri* encodes three copies of the *inlP* gene in this region. All four homologs are similar (>70 %) to InlP in *L. monocytogenes *, with the main structural difference resulting from the lack of full-length LRR6 and LRR7 regions in InlP*_Li_*, InlP*_Ls1_*, InlP*_Ls2_*, and InlP*_Ls3_*. Our findings will serve as a springboard for future evolutionary studies of internalins in the genus *Listeria* and will bolster future *in vitro* and *in vivo* studies of InlP in the context of virulence and pathogenicity.

## Supplementary Data

Supplementary material 1

Supplementary material 2
