# Supplementary figures and images for: Novel internalin P homologs in Listeria
Source: Microb Genom. 2022 Jul 29;8(7):mgen000828. doi: 10.1099/mgen.0.000828 (PMC9455699; doi:10.1099/mgen.0.000828)

**Fig. S1**

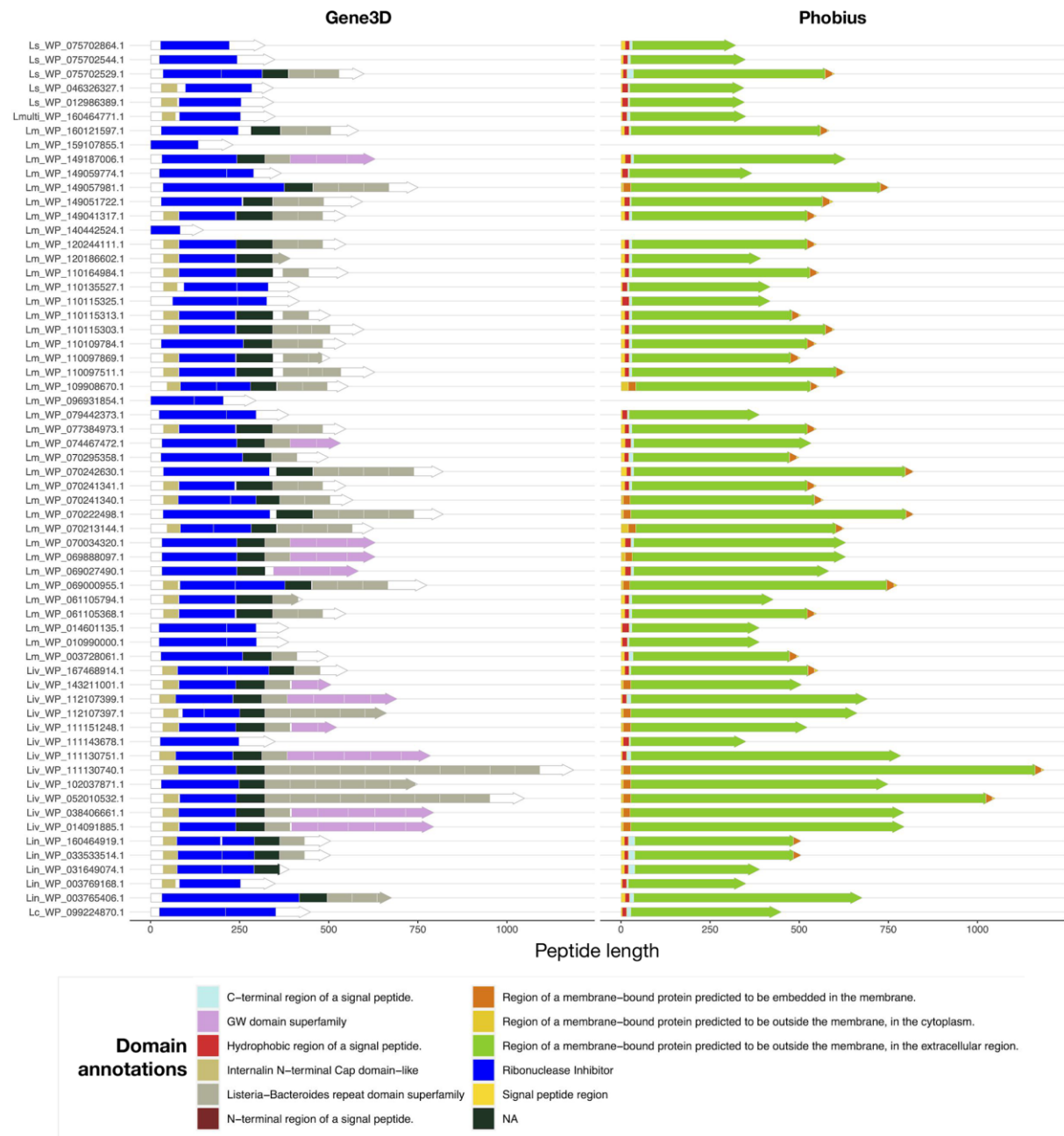

Fig. S2

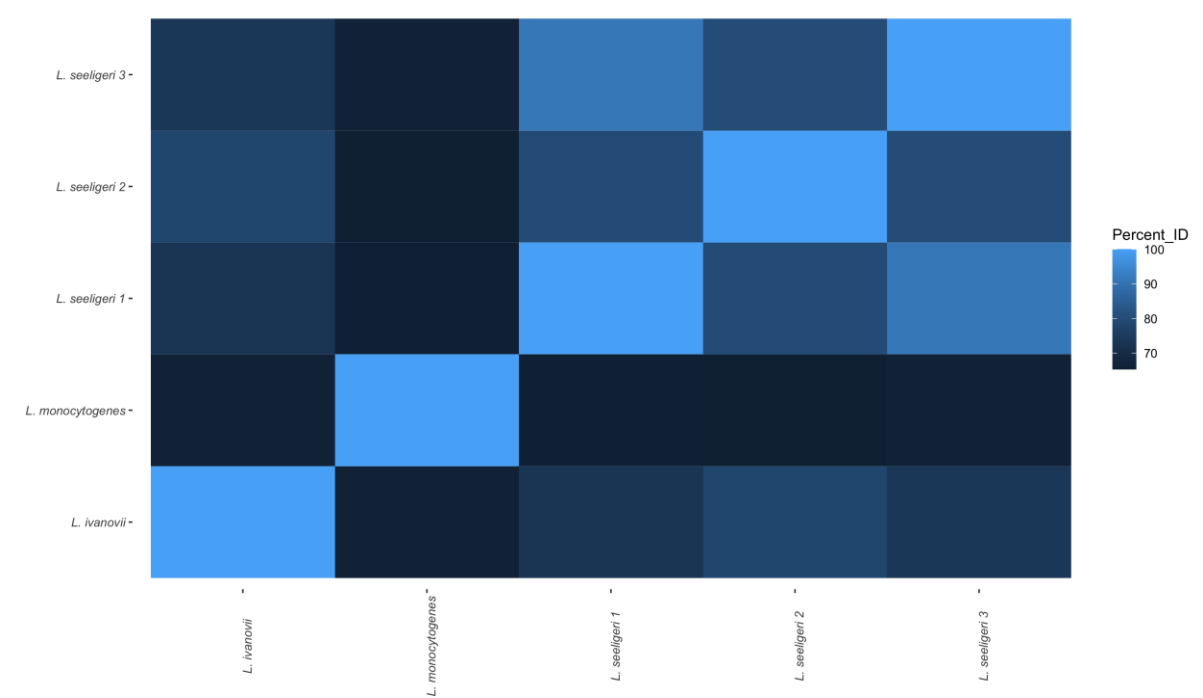

Fig. S3

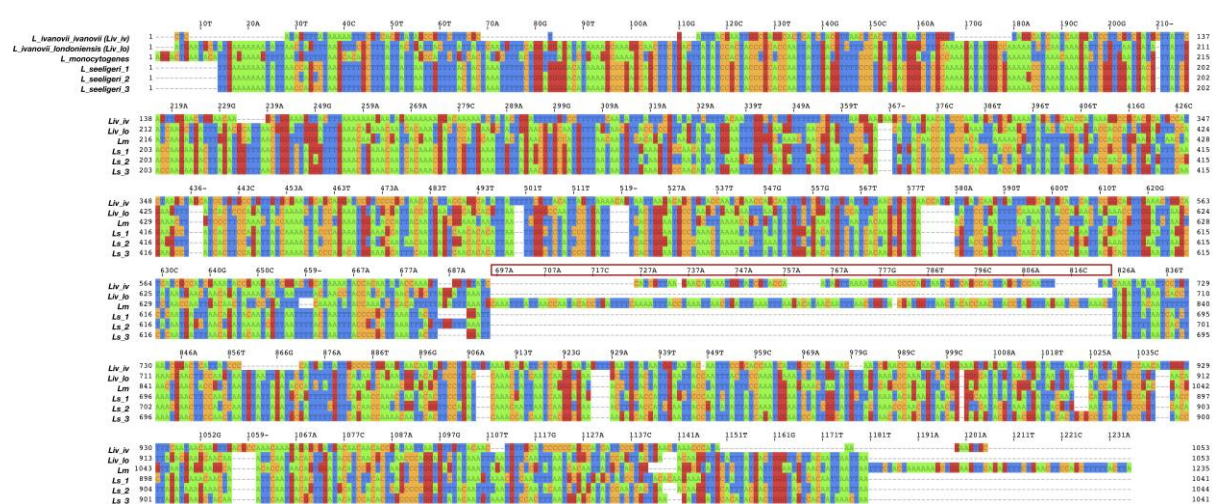

Supplement: Supplementary material 2 [file mgen-8-828-s002.pdf]
