# Supplementary material for: Novel internalin P homologs in Listeria
Source: Microb Genom. 2022 Jul 29;8(7):mgen000828. doi: 10.1099/mgen.0.000828 (PMC9455699; doi:10.1099/mgen.0.000828)
Supplement: Supplementary material 1 [file mgen-8-828-s001.pdf]

Query	Subject	Species	Lineage	PcPositive	PcIdentity	DomArch.Pfam	Isolate Source	Accession
Liv_WP_111143678.1	Lin_WP_003769168.1	Listeria innocua	Bacteria>Firmicutes	60.29	37.02	Internalin_N	Australian Dairy Farm	WP_003769168.1
Liv_WP_111143678.1	Liv_WP_014091885.1	Listeria ivanovii	Bacteria>Firmicutes	37.43	23.68	Internalin_N+<br>LRR_4+<br>LRR_adjacent+<br>Flg_new+<br>GW+<br>GW+<br>GW+<br>GW+<br>GW	Mus pahari Intestine	WP_014091885.1
Liv_WP_111143678.1	Liv_WP_052010532.1	Listeria ivanovii	Bacteria>Firmicutes	39.72	21.75	Internalin_N+<br>LRR_4+<br>LRR_adjacent+<br>Flg_new+<br>Flg_new+<br>Flg_new+<br>Flg_new+<br>Flg_new+<br>Flg_new+<br>Flg_new+<br>Flg_new+<br>Flg_new+<br>Gram_pos_anchor	NA	WP_052010532.1
Liv_WP_111143678.1	Lm_WP_061105368.1	Listeria monocytogenes	Bacteria>Firmicutes	40.58	23.36	Internalin_N+<br>LRR_4+<br>LRR_adjacent+<br>Flg_new+<br>Flg_new	Salmon Processing Facility	WP_061105368.1
Liv_WP_111143678.1	Lm_WP_061105794.1	Listeria monocytogenes	Bacteria>Firmicutes	40.29	22	Internalin_N+<br>LRR_4+<br>LRR_4+<br>LRR_adjacent+<br>Flg_new	Chicken	WP_061105794.1
Liv_WP_111143678.1	Lm_WP_069027490.1	Listeria monocytogenes	Bacteria>Firmicutes	36.85	20.95	Internalin_N+<br>LRR_8+<br>LRR_4+<br>LRR_adjacent+<br>GW+<br>GW+<br>GW	Basil	WP_069027490.1
Liv_WP_111143678.1	Lm_WP_069888097.1	Listeria monocytogenes	Bacteria>Firmicutes	37.71	21.27	Internalin_N+<br>LRR_8+<br>LRR_adjacent+<br>Flg_new+<br>GW+<br>GW+<br>GW	NA	WP_069888097.1
Liv_WP_111143678.1	Lm_WP_070034320.1	Listeria monocytogenes	Bacteria>Firmicutes	40.28	21.91	Internalin_N+<br>LRR_4+<br>LRR_adjacent+<br>Flg_new+<br>GW+<br>GW+<br>GW	Sheep Vaginal Swab	WP_070034320.1
Liv_WP_111143678.1	Lm_WP_070241341.1	Listeria monocytogenes	Bacteria>Firmicutes	40.57	22.57	Internalin_N+<br>LRR_4+<br>LRR_6+<br>LRR_4+<br>LRR_adjacent+<br>Flg_new+<br>Flg_new	Equine Liver	WP_070241341.1
Liv_WP_111143678.1	Lm_WP_070295358.1	Listeria monocytogenes	Bacteria>Firmicutes	36	21.93	Internalin_N+<br>LRR_4+<br>LRR_adjacent+<br>Flg_new	Ovine lung, liver, and heart	WP_070295358.1
Liv_WP_111143678.1	Lm_WP_074467472.1	Listeria monocytogenes	Bacteria>Firmicutes	36.85	20.95	Internalin_N+<br>LRR_8+<br>LRR_4+<br>LRR_adjacent+<br>Flg_new+<br>GW+<br>GW	Environmental	WP_074467472.1
Liv_WP_111143678.1	Ls_WP_075702864.1	Listeria seeligeri	Bacteria>Firmicutes	41.43	28.62	NA	NA	WP_075702864.1
Liv_WP_111143678.1	Lm_WP_077384973.1	Listeria monocytogenes	Bacteria>Firmicutes	40.86	22.29	Internalin_N+<br>LRR_8+<br>LRR_adjacent+<br>Flg_new+<br>Flg_new	Factory	WP_077384973.1
Liv_WP_111143678.1	Liv_WP_102037871.1	Listeria ivanovii	Bacteria>Firmicutes	38.86	22.06	Internalin_N+<br>LRR_4+<br>LRR_adjacent+<br>Flg_new+<br>Flg_new+<br>Flg_new+<br>Flg_new+<br>Flg_new+<br>Flg_new	NA	WP_102037871.1
Liv_WP_111143678.1	Lm_WP_109908670.1	Listeria monocytogenes	Bacteria>Firmicutes	31.43	24.81	Internalin_N+<br>LRR_4+<br>LRR_8+<br>LRR_adjacent+<br>Flg_new+<br>Flg_new	Human Blood	WP_109908670.1
Liv_WP_111143678.1	Lm_WP_110097511.1	Listeria monocytogenes	Bacteria>Firmicutes	40.29	22.29	Internalin_N+<br>LRR_4+<br>LRR_4+<br>LRR_adjacent+<br>LRR_adjacent+<br>Flg_new+<br>Flg_new+<br>Flg_new	Food	WP_110097511.1
Liv_WP_111143678.1	Lm_WP_110097869.1	Listeria monocytogenes	Bacteria>Firmicutes	40.29	22.29	Internalin_N+<br>LRR_4+<br>LRR_4+<br>LRR_adjacent+<br>LRR_adjacent+<br>Flg_new+<br>Flg_new	Smoked Turkey	WP_110097869.1
Liv_WP_111143678.1	Lm_WP_110115303.1	Listeria monocytogenes	Bacteria>Firmicutes	40.29	22.29	Internalin_N+<br>LRR_4+<br>LRR_4+<br>LRR_adjacent+<br>Flg_new+<br>Flg_new+<br>Flg_new	Chicken	WP_110115303.1
Liv_WP_111143678.1	Lm_WP_110115313.1	Listeria monocytogenes	Bacteria>Firmicutes	40.29	22.29	Internalin_N+<br>LRR_4+<br>LRR_4+<br>LRR_adjacent+<br>LRR_adjacent+<br>Flg_new	Meat	WP_110115313.1
Liv_WP_111143678.1	Lm_WP_110164984.1	Listeria monocytogenes	Bacteria>Firmicutes	39.71	22.57	Internalin_N+<br>LRR_6+<br>LRR_4+<br>LRR_4+<br>LRR_adjacent+<br>LRR_adjacent+<br>Flg_new+<br>Flg_new	Food	WP_110164984.1
Liv_WP_111143678.1	Liv_WP_111130740.1	Listeria ivanovii	Bacteria>Firmicutes	38.86	22.06	Internalin_N+<br>LRR_4+<br>LRR_adjacent+<br>Flg_new+<br>Flg_new+<br>Flg_new+<br>Flg_new+<br>Flg_new+<br>Flg_new+<br>Flg_new+<br>Flg_new+<br>Flg_new+<br>Flg_new+<br>Flg_new+<br>Gram_pos_anchor	Pika Intestine	WP_111130740.1
Liv_WP_111143678.1	Liv_WP_111130751.1	Listeria ivanovii	Bacteria>Firmicutes	34.29	20.2	Internalin_N+<br>LRR_8+<br>LRR_adjacent+<br>Flg_new+<br>GW+<br>GW+<br>GW+<br>GW+<br>GW	Pika Intestine	WP_111130751.1
Liv_WP_111143678.1	Liv_WP_111143678.1	Listeria ivanovii	Bacteria>Firmicutes	100	99.71	Internalin_N	Suncus murinus Intestines	WP_111143678.1
Liv_WP_111143678.1	Liv_WP_111151248.1	Listeria ivanovii	Bacteria>Firmicutes	37.43	23.68	Internalin_N+<br>LRR_4+<br>LRR_adjacent+<br>Flg_new+<br>GW+<br>GW	Pika Intestine	WP_111151248.1
Liv_WP_111143678.1	Liv_WP_112107397.1	Listeria ivanovii	Bacteria>Firmicutes	38.86	22.06	Internalin_N+<br>LRR_4+<br>LRR_adjacent+<br>Flg_new+<br>Flg_new+<br>Flg_new+<br>Flg_new+<br>Flg_new	Water	WP_112107397.1
Liv_WP_111143678.1	Liv_WP_112107399.1	Listeria ivanovii	Bacteria>Firmicutes	34.29	20.2	Internalin_N+<br>LRR_8+<br>LRR_adjacent+<br>Flg_new+<br>GW+<br>GW+<br>GW+<br>GW	Water	WP_112107399.1
Liv_WP_111143678.1	Lm_WP_120186602.1	Listeria monocytogenes	Bacteria>Firmicutes	40.57	22.29	Internalin_N+<br>LRR_6+<br>LRR_4+<br>LRR_4+<br>LRR_adjacent+<br>Flg_new	Chicken Taco	WP_120186602.1
Liv_WP_111143678.1	Liv_WP_143211001.1	Listeria ivanovii	Bacteria>Firmicutes	37.43	23.68	Internalin_N+<br>LRR_4+<br>LRR_adjacent+<br>Flg_new	Himalayan marmot Intestine	WP_143211001.1
Liv_WP_111143678.1	Lm_WP_149041317.1	Listeria monocytogenes	Bacteria>Firmicutes	40.29	22.57	Internalin_N+<br>LRR_4+<br>LRR_6+<br>LRR_4+<br>LRR_4+<br>LRR_adjacent+<br>Flg_new+<br>Flg_new	Soil	WP_149041317.1
Liv_WP_111143678.1	Lm_WP_149187006.1	Listeria monocytogenes	Bacteria>Firmicutes	40.28	22.22	Internalin_N+<br>LRR_8+<br>LRR_4+<br>LRR_adjacent+<br>Flg_new+<br>GW+<br>GW+<br>GW	Soil	WP_149187006.1
Liv_WP_111143678.1	Lmulti_WP_160464771.1	Listeria	Bacteria>Firmicutes	60.29	36.46	Internalin_N	Chicken in Russia	WP_160464771.1
Lm_WP_014601135.1	Lin_WP_003765406.1	Listeria innocua	Bacteria>Firmicutes	29.71	19.15	Internalin_N+<br>LRR_4+<br>LRR_4+<br>LRR_4+<br>LRR_4+<br>LRR_adjacent+<br>Flg_new+<br>Flg_new+<br>Flg_new	NA	WP_003765406.1
Lm_WP_014601135.1	Lm_WP_010990000.1	Listeria monocytogenes	Bacteria>Firmicutes	99.74	99.48	Internalin_N+<br>LRR_4+<br>LRR_1	NA	WP_010990000.1
Lm_WP_014601135.1	Lm_WP_014601135.1	Listeria monocytogenes	Bacteria>Firmicutes	100	99.74	NA	Environmental	WP_014601135.1
Lm_WP_014601135.1	Lin_WP_031649074.1	Listeria innocua	Bacteria>Firmicutes	31.27	20.2	Internalin_N+<br>LRR_4+<br>LRR_adjacent	NA	WP_031649074.1
Lm_WP_014601135.1	Lin_WP_033533514.1	Listeria innocua	Bacteria>Firmicutes	31.78	20.19	Internalin_N+<br>LRR_4+<br>LRR_adjacent+<br>Flg_new	Bos taurus	WP_033533514.1
Lm_WP_014601135.1	Lm_WP_070222498.1	Listeria monocytogenes	Bacteria>Firmicutes	31.27	22.36	Internalin_N+<br>LRR_4+<br>LRR_6+<br>LRR_8+<br>LRR_6+<br>LRR_adjacent+<br>Flg_new+<br>Flg_new+<br>Flg_new+<br>Flg_new	Equine Liver	WP_070222498.1
Lm_WP_014601135.1	Lm_WP_070241340.1	Listeria monocytogenes	Bacteria>Firmicutes	32.56	16.08	Internalin_N+<br>LRR_adjacent+<br>Flg_new+<br>Flg_new	Soil	WP_070241340.1
Lm_WP_014601135.1	Lm_WP_070242630.1	Listeria monocytogenes	Bacteria>Firmicutes	34.63	20.41	Internalin_N+<br>LRR_6+<br>LRR_4+<br>LRR_8+<br>LRR_6+<br>LRR_adjacent+<br>Flg_new+<br>Flg_new+<br>Flg_new+<br>Flg_new	Human	WP_070242630.1
Lm_WP_014601135.1	Ls_WP_075702529.1	Listeria seeligeri	Bacteria>Firmicutes	29.72	18.91	Internalin_N+<br>LRR_4+<br>LRR_4+<br>LRR_adjacent+<br>Flg_new+<br>Flg_new+<br>Gram_pos_anchor	NA	WP_075702529.1
Lm_WP_014601135.1	Lm_WP_079442373.1	Listeria monocytogenes	Bacteria>Firmicutes	99.23	98.45	Internalin_N+<br>LRR_4	Human Nasal Swab	WP_079442373.1
Lm_WP_014601135.1	Lm_WP_096931854.1	Listeria monocytogenes	Bacteria>Firmicutes	74.42	99.65	LRR_4+<br>LRR_1	Wild Boar Lymph Node	WP_096931854.1
Lm_WP_014601135.1	Lc_WP_099224870.1	Listeria costaricensis	Bacteria>Firmicutes	55.04	31	NA	NA	WP_099224870.1
Lm_WP_014601135.1	Lm_WP_110115325.1	Listeria monocytogenes	Bacteria>Firmicutes	99.49	92.11	Internalin_N+<br>Internalin_N+<br>LRR_4+<br>LRR_1	Meat	WP_110115325.1
Lm_WP_014601135.1	Lm_WP_110135527.1	Listeria monocytogenes	Bacteria>Firmicutes	99.49	91.63	Internalin_N+<br>LRR_8	Raw Milk	WP_110135527.1
Lm_WP_014601135.1	Lm_WP_140442524.1	Listeria monocytogenes	Bacteria>Firmicutes	36.95	91.28	LRR_1	NA	WP_140442524.1
Lm_WP_014601135.1	Lm_WP_149057981.1	Listeria monocytogenes	Bacteria>Firmicutes	31.27	22.36	Internalin_N+<br>LRR_4+<br>LRR_6+<br>LRR_8+<br>LRR_6+<br>LRR_adjacent+<br>Flg_new+<br>Flg_new+<br>Flg_new	Soil	WP_149057981.1
Lm_WP_014601135.1	Lm_WP_159107855.1	Listeria monocytogenes	Bacteria>Firmicutes	58.14	93.1	LRR_8+<br>LRR_8	Human Placenta	WP_159107855.1
Lm_WP_014601135.1	Lin_WP_160464919.1	Listeria	Bacteria>Firmicutes	31.52	19.87	Internalin_N+<br>LRR_4+<br>LRR_adjacent+<br>Flg_new	Water	WP_160464919.1
Lm_WP_014601135.1	Liv_WP_167468914.1	Listeria ivanovii	Bacteria>Firmicutes	30.23	17.06	Internalin_N+<br>LRR_4+<br>LRR_6+<br>LRR_4+<br>LRR_adjacent+<br>Flg_new	NA	WP_167468914.1
Ls_WP_012986389.1	Ls_WP_012986389.1	Listeria seeligeri	Bacteria>Firmicutes	100	100	Internalin_N	Water	WP_012986389.1
Ls_WP_012986389.1	Ls_WP_046326327.1	Listeria seeligeri	Bacteria>Firmicutes	94.51	87.57	Internalin_N+<br>LRR_8	Water	WP_046326327.1
Ls_WP_012986389.1	Lm_WP_149051722.1	Listeria monocytogenes	Bacteria>Firmicutes	41.32	21.02	Internalin_N+<br>LRR_4+<br>LRR_4+<br>LRR_4+<br>LRR_adjacent+<br>Flg_new+<br>Flg_new	Soil	WP_149051722.1
Ls_WP_012986390.1	Liv_WP_038406661.1	Listeria ivanovii	Bacteria>Firmicutes	36.6	20.74	NA	NA	WP_038406661.1
Ls_WP_012986390.1	Lm_WP_149059774.1	Listeria monocytogenes	Bacteria>Firmicutes	81.85	64.6	Internalin_N	Soil	WP_149059774.1
Ls_WP_012986390.1	Lm_WP_160121597.1	Listeria monocytogenes	Bacteria>Firmicutes	29.97	18.22	Internalin_N+<br>LRR_4+<br>LRR_4+<br>LRR_adjacent+<br>Flg_new+<br>Flg_new+<br>Gram_pos_anchor	NA	WP_160121597.1
Ls_WP_012986391.1	Lm_WP_003728061.1	Listeria monocytogenes	Bacteria>Firmicutes	39.88	21.07	Internalin_N+<br>LRR_4+<br>LRR_4+<br>LRR_4+<br>LRR_adjacent+<br>Flg_new	Food	WP_003728061.1
Ls_WP_012986391.1	Lm_WP_069000955.1	Listeria monocytogenes	Bacteria>Firmicutes	32.95	18.05	Internalin_N+<br>LRR_4+<br>LRR_4+<br>LRR_4+<br>LRR_adjacent+<br>Flg_new+<br>Flg_new+<br>Flg_new	Food Processing Plant	WP_069000955.1
Ls_WP_012986391.1	Lm_WP_070213144.1	Listeria monocytogenes	Bacteria>Firmicutes	28.9	22.35	Internalin_N+<br>LRR_4+<br>LRR_8+<br>LRR_adjacent+<br>Flg_new+<br>Flg_new+<br>Flg_new	Human	WP_070213144.1
Ls_WP_012986391.1	Ls_WP_075702544.1	Listeria seeligeri	Bacteria>Firmicutes	59.82	38.94	LRR_8	Water	WP_075702544.1
Ls_WP_012986391.1	Lm_WP_110109784.1	Listeria monocytogenes	Bacteria>Firmicutes	40.75	20.84	Internalin_N+<br>LRR_6+<br>LRR_4+<br>LRR_4+<br>LRR_adjacent+<br>Flg_new+<br>Flg_new	Food	WP_110109784.1
Ls_WP_012986391.1	Lm_WP_120244111.1	Listeria monocytogenes	Bacteria>Firmicutes	41.91	21.35	Internalin_N+<br>LRR_4+<br>LRR_4+<br>LRR_adjacent+<br>Flg_new+<br>Flg_new	Chicken sausage, wild mushrooms, garlic	WP_120244111.1

More information available on GitHub.
